# Characteristics of Soft Wheat and Tiger Nut (*Cyperus esculentus*) Composite Flour Bread

**DOI:** 10.3390/foods14020229

**Published:** 2025-01-13

**Authors:** Svitlana Nedviha, Joanna Harasym

**Affiliations:** 1 Department of Bakery and Confectionary Technology, State Biotechnological University, Alchevskih St. 44, 61002 Kharkiv, Ukraine; nedviga_sveta@ukr.net; 2 Department of Biotechnology and Food Analysis, Wroclaw University of Economics and Business, Komandorska 118/120, 53-345 Wroclaw, Poland; 3 Adaptive Food Systems Accelerator-Science Centre, Wroclaw University of Economics and Business, Komandorska 118/120, 53-345 Wroclaw, Poland

**Keywords:** tiger nut flour, composite bread, dough rheology, bioactive compounds, antioxidant activity

## Abstract

This study investigated the effects of tiger nut flour (TNF) incorporation (5–25%) on wheat-based bread characteristics. Dough rheology analysis revealed optimal gas retention at 10% TNF addition, while higher concentrations decreased dough stability. Physical analysis demonstrated that 10% TNF substitution yielded the highest specific volume (2.4 mL/g) and porosity (67.0%), with significant textural changes observed at higher concentrations. Bioactive compound analysis showed progressive increases in the total polyphenol content and antioxidant activity with increasing TNF levels, particularly in 25% TNF bread (111.31 mg TE/g dm in crumb). Storage studies over 7 days indicated that TNF incorporation affected bread staling characteristics, with 10–15% substitution levels maintaining better textural properties. The results suggest that TNF incorporation up to 15% can enhance bread’s nutritional value, while maintaining acceptable technological properties, with 10% substitution showing an optimal balance between functional benefits and bread quality.

## 1. Introduction

Bread remains a cornerstone of human nutrition, serving as a primary source of daily sustenance across diverse cultures and populations [1]. However, traditional wheat-based breads, while ubiquitous, often fall short of meeting contemporary nutritional demands, particularly regarding functional components and bioactive compounds [2,3]. This limitation has sparked extensive research into composite flour systems that can enhance bread products’ nutritional profile and functional properties while maintaining acceptable quality characteristics. Recent trends in food science and consumer preferences have driven the exploration of novel ingredients that can improve the nutritional density of bread without compromising its fundamental qualities. Among these ingredients, tiger nut (*Cyperus esculentus*), also known as chufa, has emerged as a promising candidate [4,5,6,7,8,9,10]. Tiger nuts contain various bioactive compounds, contributing to their nutritional and functional properties. The lipid content of tiger nuts is particularly notable (22.14–44.92%), while the lipid profiling of tiger nuts equals that of olive oil [11]. Oleic acid dominates at 56–85%, followed by palmitic acid (10–20%), linoleic acid (8–12%), and stearic acid (0.3–5.3%), with linolenic and palmitoleic acids present in minor amounts. The lipid structure of tiger nuts follows a specific pattern, with triacylglycerols making up 65.9% of the total lipids, while glycolipids and phospholipids contribute 5.6–6.9% and 1.4–3.1%, respectively. The sterol composition is characterised by β-sitosterol as the predominant compound (49–60 mg/100 g), accompanied by stigmasterol, campesterol, and both α- and β-tocopherols. However, their specific quantities are not detailed in the literature. The mineral content of tiger nuts is well-documented, with potassium (110.70–121.95 mg/100 g), sodium (99.95–105.6 mg/100 g), and calcium (84.05–93.75 mg/100 g) being the most abundant. The macronutrient profile of tiger nuts reveals a moderate protein content of 5.04–6.67% wet matter, and substantial carbohydrate (46.3 g/100 g) and starch (29.9% wet matter) content. Notably, the sucrose content of tiger nuts is relatively high at 13.03 g/100 g. The total fat content varies between 22 and 45% on a dry matter basis [10,11,12,13].

Research has demonstrated the versatility of tiger nut flour (TNF) in various baked products. Studies involving cookies have shown improved nutritional profiles and extended shelf life when incorporating TNF with other functional ingredients [14,15]. Moreover, recent investigations into gluten-free applications have highlighted TNF’s potential in creating products suitable for individuals with celiac disease [8,9]. The integration of TNF into bread systems presents both opportunities and challenges. While its high fibre content and water-binding properties can improve bread functionality, these characteristics may affect dough rheology and final product quality [3].

Recent studies have shown that TNF incorporation can significantly impact dough development, gas retention, and crumb structure, suggesting the need to optimise the formulation parameters [5,6] carefully. The complex interaction between TNF components and wheat flour constituents during bread making necessitates a thorough understanding of their effects on technological and functional properties [7]. Previous research has indicated that TNF’s influence on bread quality parameters varies significantly when concentrations of up to 40% are incorporated, affecting everything from dough stability to final product characteristics, specifically at higher concentrations (30–40%) [6]. However, no techno-functional data were provided, which could help explain how TNF’s presence impacted the wheat matrix.

Therefore, this study aimed to comprehensively investigate the effects of the incorporation of lower shares (5–25%) of TNF on wheat-based bread characteristics, focusing on dough development, gas retention, texture characteristics, and its change during 7 days of storage. Specifically, the research also examined the profile of the bioactive compounds in the bread during processing and storage. This investigation sought optimal TNF incorporation levels that balance improved nutritional value with acceptable bread quality characteristics.

## 2. Materials and Methods

### 2.1. Raw Materials

Both flours were purchased from a local market, namely wheat flour (Novopokrovsky Bread Products Plant, Kharkiv, Ukraine; fats 1.3%, carbohydrates 69.9% of which sugars 1%, starch 67.9%, proteins 10.8%) and TNF (JB NATURAL FOODS, S.L., Valencia, Spain), packaged in Ukraine. According to the manufacturer, the chemical composition of the TNF was presented elsewhere [5]. For this research, the flour mixtures were prepared with a 5 to 25% share of TNF.

### 2.2. Bread Preparation Method

Bread-making ingredients: 100 g wheat flour, 1.5 g salt, 1 g dry baker’s yeast, and water-based on 44% hydration (based on previous baking trials). The mixtures of wheat flour and TNF were created according to the abovementioned shares. The control sample was a bread sample with 100% wheat flour. The dough was kneaded for 6 min, using a 1000 W dough mixer, at speed level 5 (MUM58231, BOSCH, Stuttgart, Germany). The dough samples were divided into equal parts (around 110 g), shaped and placed in pre-greased moulds, then proofed at 30 ± 1 °C for 180 min in a 2 kW bread-proofing cabinet (823HO, Bartscher, Salzkotten, Germany) and baked at 200 ± 1 °C for 22 min in a steaming–convection oven (Nano, Grafen, Robakowo, Poland). Per formulation, 3 batches were produced, with 8 loaves per batch.

### 2.3. Dough Rising Characteristics

The dough rising characteristics were assessed using a rheofermentometer (Rhefermentometer 3, Chopin, Villeneuve-la-Garenne, France), which measures the pressure every 45 s in the thermostatically controlled, airtight tank that contains the dough. The device measures the total gas production (yeast action) in a direct cycle, while, during an indirect cycle, it measures gas retention, i.e., the porosity of the dough. The results from the dough development curve are denoted as: Hm, which represents the maximum development reached by the dough, correlated with the bread volume; h, which represents the maximum development reached by the dough at the end of the test; (Hm-h)/Hm [%], which represents the weakening coefficient; and T1, which represents the time required for maximum development, concerning the yeast activity. The results from the gas production curve are denoted as: H’m, which represents the maximum height of the curve; T1, which represents the time required to reach H’m; Tx, which represents the time of appearance of porosity in the dough, i.e., the time when the dough begins to release CO_2_; total volume, which represents the total volume of gas released in ml; total volume of CO_2_ lost, which represents the total volume of CO_2_, in ml, that the dough has allowed to escape during proofing; and volume of retention, which represents the volume of CO_2_, in ml, still retained within the dough at the end of the test. The dough development and gas production curves were obtained using the Chopin protocol for 3 h. Sampling occurred every 90 s. The dough to be analysed was prepared in a kneading machine (MUM58231, BOSCH, Germany), according to the recipe described in Section 2.2, and 200 g of the dough was placed in the chamber for duplicate analysis. The sample was measured in triplicate.

### 2.4. Specific Volume

The volume (*v*) of the bread loaves was measured 1 h after baking, using a 3D scanner (Matter and Form v2, Toronto, ON, Canada) and the Quickscan app v.1.1 to calculate the volume. Moreover, the change in volume was compared with freshly baked loaves by measuring the volume after 7 days of storage at (4 ± 2) °C in hermetic bags. Each sample’s weight (*w*) was also recorded and all the measurements were carried out in duplicate. The *specific volume* was calculated using the following Equation (1):

*Specific volume* [cm^3^/g] = *v/w* (1)

The sample was measured in triplicate.

### 2.5. Imaging and Cross-Section Scanning

Images of the loaves were taken using a mobile phone, a Samsung Galaxy M31 camera (a system with four lenses 64 MP (f/1.8) + 8 MP (f/2.2) + 5 MP (f/2.2) + 5 MP (f/2.4)), within a covered chamber, lit by a white lamp (Chromato-Vue^®^ C-75 UV, UVP, Upland, CA, USA). Cross-section scanning was carried out with a flat-bed scanner (X1250, Lexmark, Lexington, KY, USA) 1 h after baking and after 7 days of storage. A bread porosity analysis was performed using the Analyse particles option implemented in ImageJ (NIH, https://imagej.net/nih-image/, Bethesda, MD, USA) software on cross-section scans. The samples were measured in quadruplicate.

### 2.6. Texture Profile Analysis of Bread

The texture of the bread was assessed 1 h after baking and after 7 days of storage, using a double compression test (texture profile analysis—TPA) to penetrate 50% of the depth, at a 1 mm/s speed, with a 30 s delay between the first and second compression. The firmness (N), chewiness (N), cohesiveness, springiness, and resilience of the bread were calculated from the TPA graphic. The analysis was conducted at 20 ± 2 °C using a sample taken from two bread slices, with a thickness of 20 mm, from the loaf’s centre. The TPA test was conducted using a texture analyser, using a cylindrical probe (FC20STAV500/500, AXIS, Gdansk, Poland) with a diameter of 50 mm. The software used for the data acquisition was AXIS FM v.2_18, as mentioned previously [16]. Two loaves were used per sample and three subsamples were taken from each slice. The samples were measured in triplicate.

### 2.7. Water Activity

The water activity of the bread was measured using an AquaLab analyser (Series 3TE, Decagon Devices, Inc., Pullman, WA, USA) on the day of baking and after 7 days. The measuring capsule was filled with the entire sample cut from the slice. Each slice was sampled twice and a total measure was taken in quadruplicate.

### 2.8. Colour

The colour of the crust and the crumb was assessed 1 h after baking and after 7 days of storage using a CR-310 chroma meter (Konica Minolta, INC., Tokyo, Japan).This measurement setup was based on the methodology described by Harasym et al. in 2020 [17]. The measurements were taken eight times to determine the crust and crumb colour of the bread for each sample. The colour parameters evaluated were L*, a*, and b*.

The *whiteness index* (WI) for the bread crumb was calculated using Equation (2) and the *browning index* (BI) for the crust was determined using Equation (3) [18]:

(2) $$\mathrm{Whiteness}\mathrm{index}=100-\sqrt{{\left(100-L\right)}^{2}+a^{2}+b^{2}}$$

(3) $$\mathrm{Browning}\mathrm{index}=\frac{100\times \left(x-0.31\right)}{0.172}$$

where *x* = $\frac{a+175L}{5.645L+a-3.012b}$

The samples were measured in quadruplicate.

### 2.9. Total Polyphenol Content

A total of 1.0 g of each sample was mixed with 5 mL of an acidified methanol/water solvent (80:20 *v*/*v*) containing 1% HCl. The mixture was thoroughly mixed in a vortex machine (MX-S, ChemLand, Stargard, Poland) for 1 min. Next, the samples were agitated using a rotary shaker (MX-RD PRO, ChemLand, Stargard, Poland) at room temperature for 2 h. After the agitation, the extraction tubes were centrifuged at 3500× *g* for 15 min using a centrifuge (MPW-350, MPW Med. Instruments, Warsaw, Poland). The supernatant was then collected for further analysis. In a test tube, 20 μL of the extract was mixed with 1580 μL of distilled water. Then, 100 μL of undiluted Folin–Ciocalteu reagent was added to the mixture. The test tube was vortexed and incubated for 6 min at room temperature (25 °C). Next, 300 μL of saturated CaCO_3_ solution was added to the test tube and thoroughly mixed until a permanent blue colour was obtained. The solution was then incubated at 38 °C in a water bath (06-DK-98-IV, ChemLand, Stargard, Poland) for 30 min in the dark. Finally, the absorbance of the solution was measured at 765 nm using a spectrophotometer (SEMKO, Warsaw, Poland). The measurements were conducted using two replicates. The results of the analysis were expressed as milligrams of the gallic acid equivalent (GAE) per gram of dry matter (DM) [19,20]. The collected extract was also used for the DPPH antioxidant activity analysis. The results were carried out in quadruplicate.

### 2.10. DPPH Radical Quenching

To evaluate the antioxidant activity, the previously obtained extract was utilized. The measurement was conducted using 2,2-diphenyl-1-picrylhydrazyl as a source of free radicals. To perform the assay, a tube was prepared by adding 1000 μL of DPPH working solution. Subsequently, 34.5 μL of the sample extract was added to the tube, ensuring thorough mixing. The mixture was then incubated for 20 min in the dark at room temperature. Following the incubation period, the absorbance was measured against a blank at a wavelength of 517 nm (SEMKO, Warsaw, Poland). The measurements were conducted using two replicates. The obtained result was expressed as milligrams of the Trolox equivalent (TE) per gram of dry matter (DM) [19,20]. The results were measured in quadruplicate.

### 2.11. Reducing Sugar Content

A 1.0 g of each sample was mixed with 5 mL of distilled water for 1 min using a vortex mixer (MX-S, ChemLand, Stargard, Poland). The mixture was then agitated using a rotary shaker (MX-RD PRO, ChemLand, Stargard, Poland) at room temperature for 2 h. After extraction, the tubes were centrifuged at 3500× *g* for 15 min using a centrifuge (MPW-350, MPW MED. INSTRUMENTS, Warsaw, Poland) and the supernatant was collected. The content of the reducing sugars in the extracts was measured using a modified version of the method described by Bhajan et al. in 2023 [21], which is based on the reducing properties of sugars regarding 3,5-dinitrosalicylic acid (DNS). To measure the reducing sugar content, 1 mL of DNS reagent was mixed thoroughly with 1 mL of the sample extract. The resulting mixture was then heated in boiling water for 5 min, cooled to room temperature, and the absorbance at 535 nm was measured using a spectrophotometer (SEMKO, Warsaw, Poland). The measurements were conducted using two replicates. The content of the monosaccharides was expressed in grams of the glucose equivalent per gram of dry matter. The results were measured in quadruplicate.

### 2.12. Statistical Analyses

The results were presented as the mean values with the standard deviation. The analysis of variance (ANOVA) and multifactor ANOVA (two-way for sample and day, two-way for crumb and crust, three-way for part, sample, and solvent) were evaluated using Statgraphics Centurion software (Centurion XVII.I version, StatPoint Technologies, Inc., Warrenton, VA, USA). The ANOVA was performed with previously checked data regarding normality, using a *p*-value < 0.05 significance level.

## 3. Results and Discussion

### 3.1. Dough Characteristics

Table 1 presents the results for the dough rising, gas formation, and gas release from the composite bread dough.

The control sample showed the highest maximum rise (29.8 mm), and as the TNF share increased, the values decreased, resulting in the mixture with 20% TNF showing the lowest Hm. The same trend is noted in regard to the final height of the dough (h). The dough weakening values calculated as (Hm-h)/Hm have higher values than the control sample for the 25% and 20% TNF mixture and the highest value for the mixture with 5% TNF.

In comparison, the control sample had a dough weakening value of 4.9% and the mixture with 10% tiger nut flour showed no difference between the Hm and h, indicating a decrease in dough weakening at this level. The control sample took 158.3 min to rise, the mixture with 10% TNF took the longest time, and the mixture with 25% TNF achieved the fastest at 103.0 min.

The gas formation (H′m) increased with higher TNF content, peaking at 25% TNF, with an increase of 22.8% over the control. However, the retention rate decreased (−4.1%) as the gas retention capacity of the dough weakened. The value of the mixture with 25% TNF showed the highest total volume and the highest gas loss, indicating poor gas retention at this amount of TNF addition, while the control sample had minimal CO_2_ loss. Mixtures with 5% and 10% TNF had improved gas retention, by 4.6% and 23.4%. The excessive addition of TNF worsens the gas retention and dough stability. Studies involving wheat and buckwheat flours have shown a notable increase in both the volume of CO_2_ lost and the volume of CO_2_ retained within the dough [22], which was related to the intensified fermentation process, probably due to the higher amount of simple (reducing) sugars in buckwheat [22].

Similar results were obtained in studies involving the addition of olive pomace to bread dough [23]. The observed decrease in dough height, reduction in gas formation, and increase in gas retention are directly related to the absence of gluten and the high fibre content in olive pomace. The high fibre content in olive pomace weakens the gluten network structure and reduces its elasticity. This results in a denser dough, which affects its ability to expand during fermentation [23].

Similar results were reported by researchers incorporating other protein and fat-containing raw materials, such as milk thistle seed (MTF) or legume flour [24,25] or flax seed meal [26].

For TNF, the gas production analysis showed that at the 10% substitution level, the mixture achieved the highest total gas volume (1694 mL) and optimal gas retention characteristics. However, increasing the TNF levels above 15% led to a decrease in the gas retention capability of the dough, with the total gas volume dropping to 1360 mL at 20% TNF. Similar trends were observed with MTF, with a loss of significantly higher amounts of CO_2_ compared to the control flour. Still, interestingly, the dough incorporating MTF maintained the gas retention volume at approximately the same level as the control. The MTF studies showed that the total gas volume of the dough remained relatively stable with a substitution of up to 15% MTF. It was also shown that germinated legume flours generally enhanced CO_2_ production during fermentation, likely due to the increased availability of mono- and disaccharides that served as substrates for yeast fermentation [25]. The maximum height of the gaseous output showed distinct patterns across the different flour types.

For TNF, this parameter peaked at the addition of 5% TNF (68.7 mm) and gradually decreased with higher substitution levels. MTF showed similar behaviour, but maintained better gas production characteristics at higher substitution levels. The gas retention coefficient was particularly interesting, TNF showed values ranging from 76.55% to 86.45% depending on the concentration. MTF maintained a retention coefficient between 65 and 80%, demonstrating how different non-cereal flours can significantly impact dough fermentation characteristics. The decreased gas retention at higher substitution levels across all non-cereal flours can be explained by the impact of their proteolytic activity on the gluten network, as well as gluten dilution. Protease enzymes can hydrolyse peptide bonds, promoting partial denaturation of the protein network and, thus, reducing the dough’s ability to enclose air. This aligns with observations in both TNF and flaxseed studies, where higher substitution levels led to weakened gluten networks [26].

### 3.2. Quality Characteristics of the Composite Bread

The external appearance and cross-sections of the composite breads are shown in Supplementary Figures S1 and S2.

Table 2 shows the effect of adding TNF at different concentrations (0% to 25%) on the volume, weight, specific volume, porosity, and water activity of the composite bread measured on the day of baking (0) and again after 7 days (7). The results for each day are analysed separately.

The loaf volumes ranged from 243.6 mL (10% tiger nut flour) to 186.8 mL (25% TNF), decreasing with higher TNF content. Flaxseed flour shows similar effects, with 10–15% inclusion reducing the loaf volume by 6.7–11.4% [26]. TNF caused a greater volume reduction (18% at 10–15% substitution) compared to flaxseed flour (11.4%) [26], due to the stronger impact of TNF on the gas retention and fibre content of the bread.

The specific volumes varied from 2.4 mL/g (10% substitution) to 1.8 mL/g (25% substitution), with the control and 15% mixture displaying identical volumes. At 20% TNF, the volume decreased to 1.9 mL/g. Small particles increase the dough density by filling gluten network spaces [27], while the fat and fibre content in TNF further reduces the specific volume [28,29]. The highest porosity was found in the 10% and 15% TNF samples (67.0% and 67.6%), with the results for 5% TNF similar to the control, while 20% and 25% TNF had lower porosity. The water activity increased with higher TNF substitution levels, reaching a peak of 0.978 for 10% and 15% TNF, which can be attributed to the hygroscopic properties of TNF, confirming superior water retention. An increase in the water activity in the bread crumb is observed with the addition of flaxseed flour, primarily due to the presence of flaxseed gum. Flaxseed gum, a sticky substance, increases the water-holding capacity of the dough [26]. A slight increase in water activity is also present in bread with low carbohydrate and high-fat content [30].

When analysing the data some fibre integration effects can be observed, as incorporating TNF fibre (35.42 ± 0.09 g/100 g) created notable modifications. The specific volume peaked at 10% TNF (2.4 ± 0.1 mL/g), before declining, while the porosity increased from 62.3 ± 5.0% (control) to 67.0 ± 5.0% (10% TNF). The crumb hardness showed an increase above 15% TNF and the fibre–matrix interaction presented a critical concentration threshold at 15% TNF [31,32]. The fibre interaction also impacted the water-binding capacity, as the water distribution patterns showed concentration-dependent behaviour. The water activity ranged from 0.9715 ± 0.001 to 0.9675 ± 0.001 and the initial water absorption increased by 0.8% per 1% TNF addition. After 7 days of storage, the water activity patterns remained relatively stable for samples with 5–15% TNF. These modifications influence both the initial bread characteristics and the storage stability, with optimal water distribution observed in the 10–15% TNF range, where sufficient hydration is maintained for both gluten network development and fibre hydration [33].

The texture parameters of the wheat/tiger nut composite bread are presented in Table 3.

The hardness values range from 5.48 N in the 10% TNF substituted sample to 11.00 N in the 25% TNF substituted sample. A comparable study involved the incorporation of TNF into bread formulations. Adding 20% TNF results in a 52.2% reduction in hardness, while the inclusion of 40% TNF leads to a 61.5% decrease. In contrast, adding hydrocolloid xanthan gum reduces the hardness to a lesser extent, as this addition enhances the structure and viscosity of the dough [34]. The control sample shows moderate hardness at 8.14 N.

The control sample shows the highest cohesiveness (0.781), while the 20% and 25% TNF substituted samples show the lowest (0.585 and 0.572, respectively), and the result for the mixture with 5% TNF is close to the control. This suggests that higher amounts of TNF lead to decreased cohesion, affecting the bread structure.

The springiness index in all the mixtures decreases relative to the control sample (0.845). But the springiness of the mixtures with 5%, 10%, and 15% TNF decrease to a lesser extent (0.777–0.719) than those with 20% and 25% TNF (0.695–0.671). The chewiness is highest in the sample with the 5% TNF replacement (5.773 N) and the lowest in the sample with 10% TNF replacement (2.620 N). The control shows moderate chewiness (5.345 N), while adding TNF generally reduces chewiness, possibly due to decreased cohesion.

The resilience of the control sample (0.50) was the highest and, in the TNF mixtures, it decreased with a higher content of TNF. The increase in bread hardness, along with the decrease in elasticity, cohesiveness, and chewiness, is associated with the high dietary fibre content of TNF and the reduced specific volume. Elevated fibre levels can alter the textural properties of bread, leading to a firmer structure. A diminished specific volume also limits the dough’s aeration and gas retention capabilities, contributing to the overall hardness of the bread [35].

The high fibre content of TNF (35.42 ± 0.09 g/100 g) interferes with gluten development by creating physical barriers between the protein molecules, thereby limiting their ability to form continuous networks [28]. This interference becomes particularly pronounced with TNF substitutions above 15%, in alignment with the observed decreases in dough stability and the gas retention capacity. Fibre integration effects manifest through both physical and chemical mechanisms. TNF’s dietary fibre components, primarily cellulose and hemicellulose, form a secondary structural network that competes with gluten proteins for available water [31]. The fibre network also provides additional gas nucleation sites during fermentation, influencing the final crumb structure of the bread [32]. The water-binding capacity represents a critical mediating factor regarding these structural modifications. Compared to wheat flour, TNF’s higher water absorption capacity results in modified water distribution patterns throughout the matrix [33].

These structural modifications directly relate to the functional properties of bread, with optimal results observed at 10% TNF substitution, where sufficient gluten network integrity is maintained. At the same time, beneficial fibre integration is achieved [34]. Higher substitution levels (>15%) lead to excessive structural disruption, manifesting as a decreased specific volume and altered textural properties.

The parameters of the crust and crumb colour of the bread on the day of baking and after 7 days are presented in Table 4.

The control bread showed the highest L* values (crumb: 73.18, crust: 77.14), while the TNF mixtures ranged from 68.74–65.14 (crumb) and 69.48–54.95 (crust). The highest a* values were found for the 20–25% TNF addition (crumb: 2.5) versus the control (0.3). The b* values increased with the TNF amount for both the crumb (highest at 20%) and the crust (highest at 10%). Similar colour trends occur with chickpea and flaxseed flour additions [27,36]. The control showed the highest L* values (crumb: 75.09, crust: 77.44). The highest a* values were found for the 20% (crumb: 2.3) and 25% (crust: 14.3) TNF mixtures. In regard to the b* values, the 20% TNF mixture achieved the highest peak for the crumb, while the 5% TNF mixture achieved the highest peak for the crust. Darker crusts resulted from the enhanced Maillard reaction, due to amino acids and reducing sugars presence [28].

**Figure 1:**
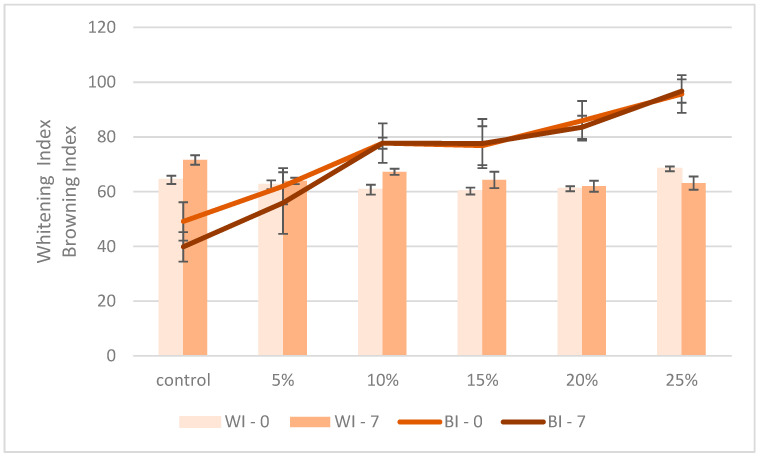
Whitening index (WI) and browning index (BI) of the samples on the day of baking and after 7 days of storage.

The whitening and browning indices are presented in Figure 1.

Figure 1 illustrates the colour development in TNF composite bread using whitening and browning indexes. The BI shows a distinct pattern, increasing from 40 in control to 80 for the 10% TNF concentration and reaching 95 in the 25% TNF sample. This reflects the enhanced Maillard reaction, due to a higher protein and reducing sugar content. The WI remains relatively stable between 60 and 70 across the formulations, indicating that TNF primarily affects crust browning rather than crumb lightness. Both indices demonstrate good stability during 7-day storage, with minimal value changes. The intersection of the BI curves for the 10% TNF concentration suggests an optimal level for balanced colour development, in alignment with the other quality parameters noted in this study.

### 3.3. Compositional Characteristics of the Composite Bread

Select compositional traits of the composite bread’s crust and crumb, expressed as reducing sugars, the total polyphenol content, and antioxidant activity, are presented below in Table 5, Table 6 and Table 7.

The reducing sugar content varied between the crust and the crumb, with the lowest crust values for the 5% TNF mixture and the highest for the 25% TNF mixture. The control sample showed uniform distribution between the crust and the crumb. The 10% and 15% TNF mixtures had similar crust values, while the 5% and 10% TNF mixtures were indistinguishable regarding the crumb. The bread crumb showed the highest reducing sugars (13.29 ± 0.52 mg Glu/g d.m.) in the 15% TNF mixture versus the control (7.07± 0.15 mg Glu/g d.m.). TNF’s inherent reducing sugar content (6.7 mg/g DM) [5] directly contributes to the elevated total sugar level in the final products.

The polyphenol content varied distinctly between the bread crumb and the crust, with the 15% TNF mixture showing the highest levels in the crumb and the 20% TNF mixture showing the highest levels in the crust. The 5% TNF mixture demonstrated the lowest polyphenol content regarding both bread parts. The crust polyphenol content levels were similar across the 15%, 20%, and 25% TNF mixtures. 

TNF is rich phenolic profile and high antioxidant activity, crucial for neutralizing free radicals [6]. Similar antioxidant enhancement occurs with the addition of flaxseed flour, which is attributed to its high polyphenol content [36]. These findings demonstrate TNF’s potential as a functional ingredient for enhancing bread’s antioxidant properties, with optimal benefits observed at 15–20% incorporation levels.

The results on the antioxidant activity versus DPPH in the crust and crumb of the composite bread are presented in Table 7.

The antioxidant activity of the bread crumb and crust in the mixtures increases with the amount of TNF. The highest result in terms of the antioxidant activity in the crust and the bread crumb was found for the mixture with 25% TNF, both about extraction using ethanol and water. A significant difference in the antioxidant activity of the crumb and crust of the bread is present in the mixtures with 5% and 10% TNF.

The bread crumb from the mixture with 5% TNF, in the extraction with ethanol, has a lower result (11.9 TE mg/g db) than the extraction with water (23.81 TE mg/g db). In the bread crust, on the contrary, the extract with water (6.49 TE mg/g db) has a lower result than with ethanol (13.80 TE mg/g db). The mixture with 10% tiger nut flourTNF shows the same trend. The lowest result found in the bread crumb and the crust, with the ethanol extract, is for the mixture with 5% TNF. With the water extract, the lowest result found in the bread crumb is for the control sample, and in the crust, the mixture with 5% TNF. These findings imply that adding TNF to bread formulations can enhance the functional properties of bread, particularly in the crust, which retains high antioxidant capacity even after baking. The same dependence is present in bread with added flaxseed flour [36]. An increase in radical activity DPPH was also observed when adding sesame seeds to cookies, due to the antioxidant content in the raw material [30].

Integrating TNF (TNF) into wheat bread systems reveals a complex interplay between the structural properties and the bioactive compound content. Statistical analysis demonstrates that optimal bread quality parameters occur at different TNF concentrations than that required for the maximal bioactive compound levels, presenting a significant challenge for product optimization. The peak in bread quality parameters (specific volume, porosity, and texture) occurs at 10–15% TNF substitution, aligning with previous findings by Aguilar et al. [8], who reported optimal gluten network formation and gas retention at similar concentrations. This phenomenon can be attributed to the balance between gluten dilution and fibre integration effects. However, the maximum bioactive compound content and antioxidant activity are observed at higher TNF levels (20–25%), creating a clear technological trade-off.

Statistical analysis reveals strong correlations between reducing sugars and the TPC results in the crust (r = 0.83, *p* < 0.01), suggesting enhanced Maillard reaction products at higher TNF concentrations, since these also give a positive response in the total phenolics assay. This aligns with Özcan’s findings [6], showing increased phenolic compounds in TNF-enriched breads. The different optimal ranges for the quality versus the bioactive properties can be explained through the competing mechanisms of structure formation and compound retention, as described by Martín-Esparza et al. [34]. The observed dichotomy between the structural and functional properties follows similar patterns reported regarding other composite flour systems. Wang et al. [7] demonstrated comparable trade-offs in fibre-enriched breads, where the optimal texture occurred at lower substitution levels than those required for maximum nutritional benefits.

Interestingly, the correlation between TPC and antioxidant activity (r = 0.79, *p* < 0.05) in the crust suggests that Maillard reaction products contribute significantly to the antioxidant properties, as previously reported by Guan et al. [7]. This indicates that higher TNF concentrations may compromise the bread structure but enhance the functional properties through the direct compound contribution and reaction product formation during baking. Future research could explore processing modifications or additional ingredients that maintain the structural integrity of bread at higher TNF concentrations to resolve this quality–functionality trade-off [37,38]. 

## 4. Conclusions

TNF incorporation significantly impacted the bread properties across different substitution levels. The 10% TNF mixture showed optimal dough characteristics, with enhanced gas retention and fermentation stability. While the 5–15% TNF mixtures maintained acceptable dough properties, while TNF levels above 20% altered the dough behaviour due to gluten dilution. Moderate TNF (10–15%) levels produced superior specific volume and porosity in the dough, but higher levels led to a denser crumb and an altered texture. Higher TNF levels created darker, reddish-brown products through increased Maillard reactions. The bioactive compounds increased progressively, peaking at the 25% TNF substitution level. The 10–15% TNF mixtures had improved storage stability over 7 days, while the mixtures with higher concentrations of TNF showed potential shelf-life benefits. This indicates that 10–15% TNF balances functionality and quality. Future research could explore TNF modification regarding higher incorporation levels.

## Figures and Tables

**Table 1:** Dough rise and gas production and gas release in composite bread doughs.

Phase	Parameters	Control	5%	10%	15%	20%	25%
Dough development	Hm [mm]	29.8 ± 0.8 ^e^	27.3 ± 1.3 ^d^	21.5 ± 0.2 ^c^	19.7 ± 0.4 ^ab^	19.2 ± 0.2 ^a^	21.2 ± 0.1 ^bc^
	h [mm]	28.4 ± 0.5 ^e^	23.5 ± 0.3 ^d^	21.5 ± 0.2 ^c^	19.2 ± 0.4 ^ab^	18.5 ± 0.1 ^a^	19.7 ± 0.1 ^b^
	(Hm-h)/Hm [%]	4.9 ± 1.0 ^bc^	13.7 ± 3.2 ^d^	0.0 ± 0.0 ^a^	2.3 ± 0.4 ^ab^	4.9 ± 2.5 ^bc^	7.1 ± 0.6 ^c^
	T1 [min]	158.3 ± 9.5 ^cd^	132.5 ± 7.8 ^b^	180.0 ± 0.0 ^e^	160.0 ± 0.7 ^d^	145.8 ± 2.5 ^c^	103.0 ± 3.5 ^a^
Gas production	H′m [mm]	32.0 ± 2.5 ^b^	33.6 ± 1.3 ^b^	30.0 ± 2.1 ^ab^	27.3 ± 0.7 ^a^	32.3 ± 0.3 ^b^	39.3 ± 0.5 ^c^
	T′1 [min]	177.0 ± 0.0 ^c^	177.5 ± 3.5 ^c^	170.5 ± 6.4 ^bc^	179.0 ± 1.4 ^c^	161.0 ± 2.8 ^b^	120.5 ± 12.0 ^a^
	Tx [min]	146.0 ± 2.8 ^c^	126.0 ± 2.8 ^b^	147.5 ± 9.2 ^c^	146.0 ± 7.1 ^c^	126.0 ± 4.2 ^b^	80.0 ± 1.4 ^a^
	Total volume [mL]	652.5 ± 65.8 ^ab^	817.5 ± 40.3 ^d^	694.0 ± 42.4 ^bc^	603.5 ± 3.5 ^a^	777.0 ± 5.7 ^cd^	966.5 ± 4.9 ^e^
	Volume of CO_2_ lost [mL]	6.0 ± 1.4 ^a^	19.5 ± 0.7 ^b^	17.5 ± 2.1 ^b^	8.5 ± 0.7 ^a^	23.5 ± 3.5 ^b^	48.5 ± 6.4 ^c^
	Volume of retention [mL]	646.5 ± 64.3 ^a^	798.0 ± 39.6 ^c^	676.5 ± 44.5 ^ab^	595.5 ± 2.1 ^a^	756.0 ± 7.1 ^bc^	917.5 ± 10.6 ^d^
	Retention coefficient [%]	99.1 ± 0.0 ^c^	97.6 ± 0.0 ^b^	98.3 ± 0.6 ^bc^	98.6 ± 0.1 ^c^	97.4 ± 0.1 ^b^	95.0 ± 0.6 ^a^

Notes: 5–25% tiger nut flour (TNF) addition; mean values with different lower case letters imply significant differences between the means in the rows at *p* < 0.05. Hm: maximum development reached by the dough; T1: time required for maximum development; T2–T′2: relative stability time at the maximum point; H′m: maximum height of the gas production curve; T1: time required to reach H′m; Tx: time when the dough begins to release CO_2_; total volume: total volume of gas released in mL; total volume of CO_2_ lost: total volume of CO_2_ lost, in mL, that the dough has allowed to escape during proofing; volume of retention: volume of CO_2_, in mL, still retained within the dough at the end of the test.

**Table 2:** The volume, weight, specific volume, porosity, and water activity of wheat/tiger nut composite bread on the baking day and 7 days after.

Sample	Days	Volume [mL]	Weight [g]	Specific Volume [mL/g]	Porosity [%]	Water Activity
Control	0	236.3 ± 0.5 ^de^	104.9 ± 0.4 ^ab^	2.3 ± 0.1 ^c^	62.3 ± 5.0 ^bc^	0.972 ± 0.001 ^b^
	7	233.2 ± 2.5 ^CD^	101.8 ± 0.4 ^BC^	2.3 ± 0.0 ^C^	62.2 ± 10.3 ^B^	0.976 ± 0.001 ^B^
5%	0	207.0 ± 2.5 ^c^	104.7 ± 0.5 ^ab^	2.0 ± 0.0 ^b^	64.3 ± 9.3 ^cd^	0.974 ± 0.001 ^b^
	7	204.1 ± 4.4 ^B^	99.0 ± 0.5 ^A^	2.1 ± 0.1 ^B^	59.1 ± 3.8 ^B^	0.974 ± 0.001 ^BC^
10%	0	243.6 ± 3.2 ^e^	104.0 ± 0.3 ^a^	2.4 ± 0.1 ^c^	67.0 ± 5.0 ^d^	0.978 ± 0.001 ^c^
	7	239.6 ± 4.8 ^DE^	99.6 ± 1.8 ^AB^	2.4 ± 0.0 ^C^	53.3 ± 1.9 ^A^	0.970 ± 0.001 ^AB^
15%	0	232.6 ± 4.2 ^d^	103.7 ± 0.6 ^a^	2.3 ± 0.1 ^c^	67.6 ± 8.2 ^d^	0.978 ± 0.001 ^c^
	7	230.7 ± 3.9 ^C^	100.3 ± 0.5 ^ABC^	2.3 ± 0.0 ^C^	54.4 ± 2.9 ^A^	0.970 ± 0.004 ^AB^
20%	0	195.8 ± 2.3 ^b^	105.8 ± 0.8 ^b^	1.9 ± 0.1 ^a^	59.7 ± 8.4 ^b^	0.969 ± 0.001 ^a^
	7	195.4 ± 1.7 ^A^	100.7 ± 1.5 ^ABC^	2.0 ± 0.1 ^AB^	54.4 ± 2.6 ^A^	0.968 ± 0.003 ^A^
25%	0	186.8 ± 4.2 ^a^	104.9 ± 0.1 ^ab^	1.8 ± 0.0 ^a^	48.0 ± 2.2 ^a^	0.9675 ± 0.001 ^a^
	7	188.5 ± 2.9 ^A^	102.2 ± 0.3 ^C^	1.9 ± 0.1 ^A^	55.0 ± 1.5 ^A^	0.968 ± 0.004 ^A^
Sample		***	*	***	***	***
Days		ns	***	*	***	*
Sample × days		ns	ns	ns	***	**

Notes: 5–25% TNF addition; mean values with different lower case (0 days) and upper case (7 days) letters imply significant differences between the means in the rows at *p* < 0.05. Second order interaction analysis *—*p* < 0.05; **—*p* < 0.01; ***—p < 0.001, ns—non-significant.

**Table 3:** Texture parameters of wheat/tiger nut composite bread loaves on the baking day and 7 days after.

Sample	Day	Hardness [N]	Cohesiveness	Springiness	Chewiness [N]	Resilience
Control	0	8.14 ± 1.20 ^bc^	0.781 ± 0.045 ^c^	0.845 ± 0.065 ^c^	5.345 ± 0.723 ^c^	0.50 ± 0.04 ^e^
	7	23.99 ± 5.27 ^B^	0.598 ± 0.198 ^B^	0.727 ± 0.057 ^D^	10.137 ± 3.417 ^C^	0.37 ± 0.10 ^AB^
5%	0	10.14 ± 2.25 ^cd^	0.734 ± 0.098 ^c^	0.777 ± 0.101 ^bc^	5.773 ± 1.499 ^c^	0.42 ± 0.05 ^a^
	7	27.56 ± 5.73 ^B^	0.439 ± 0.030 ^A^	0.687 ± 0.047 ^CD^	8.285 ± 1.730 ^BC^	0.28 ± 0.03 ^A^
10%	0	5.48 ± 1.40 ^a^	0.668 ± 0.030 ^b^	0.721 ± 0.031 ^ab^	2.620 ± 0.564 ^a^	0.40 ± 0.03 ^ab^
	7	14.56 ± 4.63 ^A^	0.494 ± 0.175 ^AB^	0.685 ± 0.081 ^CD^	4.684 ± 1.252 ^A^	0.43 ± 0.26 ^B^
15%	0	6.38 ± 1.93 ^ab^	0.646 ± 0.037 ^b^	0.719 ± 0.116 ^ab^	2.904 ± 0.851 ^a^	0.37 ± 0.04 ^bc^
	7	17.41 ± 3.92 ^A^	0.428 ± 0.029 ^A^	0.640 ± 0.047 ^BC^	4.839 ± 1.480 ^A^	0.28 ± 0.03 ^A^
20%	0	10.08 ± 2.04 ^cd^	0.585 ± 0.013 ^a^	0.695 ± 0.054 ^ab^	4.094 ± 0.897 ^b^	0.32 ± 0.01 ^cd^
	7	25.44 ± 1.58 ^B^	0.416 ± 0.025 ^A^	0.546 ± 0.114 ^A^	5.744 ± 1.094 ^A^	0.28 ± 0.03 ^A^
25%	0	11.00 ± 1.38 ^d^	0.572 ± 0.025 ^a^	0.671 ± 0.077 ^a^	4.208 ± 0.600 ^b^	0.34 ± 0.03 ^d^
	7	26.19 ± 2.03 ^B^	0.399 ± 0.028 ^A^	0.599 ± 0.029 ^AB^	6.288 ± 0.936 ^AB^	0.28 ± 0.03 ^A^
Sample		***	***	***	***	***
Day		***	***	***	***	***
Sample × day		*	ns	ns	ns	ns

Notes: 5–25% TNF addition; mean values with different lower case (0 days) and upper case (7 days) letters imply significant differences between the means in the rows at *p* < 0.05. Second-order interaction analysis *—*p* < 0.05; ***—*p* < 0.001, ns—non-significant.

**Table 4:** Bread loaves’ crust and crumb colour parameters on the baking day and after 7 days.

Sample	Days	Crumb			Crust		
		L*	a*	b*	L*	a*	b*
Control	0	73.18 ± 1.17 ^d^	0.3 ± 0.1 ^a^	16.8 ± 0.3 ^a^	77.14 ± 2.59 ^e^	5.8 ± 1.0 ^a^	27.9 ± 2.3 ^a^
	7	75.09 ± 1.77 ^C^	0.3 ± 0.1 ^A^	13.6 ± 0.5 ^A^	77.44 ± 1.33 ^E^	5.2 ± 0.8 ^A^	23.4 ± 2.3 ^A^
5%	0	68.74 ± 1.65 ^c^	1.5 ± 0.3 ^b^	17.1 ± 0.4 ^ab^	69.48 ± 2.21 ^d^	9.3 ± 1.0 ^b^	28.9 ± 1.5 ^a^
	7	70.69 ± 1.16 ^B^	1.0 ± 0.2 ^B^	14.5 ± 0.9 ^B^	69.05 ± 2.97 ^D^	8.5 ± 1.8 ^B^	26.2 ± 3.0 ^B^
10%	0	66.83 ± 2.03 ^b^	1.7 ± 0.3 ^b^	17.3 ± 0.8 ^ab^	64.14 ± 0.79 ^c^	12.0 ± 0.2 ^d^	31.2 ± 0.4 ^b^
	7	67.46 ± 1.24 ^A^	1.4 ± 0.3 ^C^	15.5 ± 0.5 ^C^	63.15 ± 2.16 ^C^	12.0 ± 0.9 ^C^	30.6 ± 1.1 ^C^
15%	0	64.89 ± 1.75 ^a^	2.2 ± 0.5 ^c^	17.4 ± 0.6 ^ab^	63.70 ± 2.16 ^c^	11.9 ± 1.1 ^c^	30.6 ± 1.0 ^b^
	7	67.74 ± 2.99 ^A^	1.7 ± 0.6 ^D^	15.1 ± 0.8 ^BC^	62.83 ± 3.21 ^C^	11.9 ± 1.3 ^C^	30.3 ± 1.0 ^C^
20%	0	64.51 ± 1.39 ^a^	2.5 ± 0.4 ^cd^	17.7 ± 0.5 ^b^	59.46 ± 2.52 ^b^	13.3 ± 0.9 ^e^	30.7 ± 0.9 ^b^
	7	66.04 ± 2.13 ^A^	2.3 ± 0.3 ^E^	16.8 ± 0.4 ^D^	59.78 ± 1.90 ^B^	12.8 ± 0.6 ^C^	30.3 ± 1.1 ^C^
25%	0	65.15 ± 1.10 ^a^	2.5 ± 0.4 ^d^	17.1 ± 1.2 ^ab^	54.95 ± 3.15 ^a^	14.3 ± 1.0 ^f^	30.4 ± 0.6 ^b^
	7	66.69 ± 2.19 ^A^	2.1 ± 0.3 ^E^	15.6 ± 1.1 ^C^	54.23 ± 2.20 ^A^	14.3 ± 0.8 ^D^	30.2 ± 0.8 ^C^
Sample		***	***	***	***	***	***
Day		***	***	***	ns	ns	***
Sample × day		ns	ns	***	ns	ns	***

Notes: 5–25% TNF addition; mean values with different lower case (0 days) and upper case (7 days) letters imply significant differences between the means in the rows at *p* < 0.05. Second-order interaction analysis ***—*p* < 0.001, ns—non-significant.

**Table 5:** Reducing sugar content in the crust and crumb of the composite breads.

Sample	Crust mg Glu/g DM	Crumb mg Glu/g DM
Control	7.13 ± 0.04 ^b^	7.07 ± 0.15 ^a^
5%	3.21 ± 0.13 ^a^	7.93 ± 0.11 ^ab^
10%	9.43 ± 0.23 ^c^	8.55 ± 0.10 ^b^
15%	9.64 ± 0.02 ^c^	13.29 ± 0.52 ^d^
20%	10.89 ± 0.37 ^d^	10.59 ± 0.66 ^c^
25%	13.36 ± 0.45 ^e^	9.67 ± 0.61 ^c^

Notes: 5–25% TNF addition; Glu—glucose equivalent; DM—dry matter, mean values with different lower case letters imply significant differences between the means in the column at *p* < 0.05.

**Table 6:** Total polyphenol content in the crust and crumb of the composite bread.

Sample	Crumb	Crust
	mg GA/100 g DM	
Control	5.16 ± 0.03 ^ab^	2.86 ± 2.02 ^a^
5%	4.29 ± 0.78 ^a^	2.75 ± 1.41 ^a^
10%	6.16 ± 1.74 ^ab^	3.57 ± 0.01 ^a^
15%	7.88 ± 0.26 ^b^	4.10 ± 0.30 ^a^
20%	7.07 ± 2.26 ^ab^	4.32 ± 0.01 ^a^
25%	5.85 ± 0.11 ^ab^	4.24 ± 0.08 ^a^

Notes: 5–25% TNF addition; GA—gallic acid equivalent, DM—dry matter, mean values with different lower case letters imply significant differences between the means in the columns at *p* < 0.05.

**Table 7:** Antioxidant activity vs. DPPH in the crust and crumb of the composite bread.

Sample	Solvent	TE mg/g DM	
		Crumb	Crust
Control	E	15.31 ± 4.33 ^a^	31.41 ± 3.30 ^b^
	W	15.68 ± 1.85 ^a^	72.48 ± 1.92 ^d^
5%	E	11.90 ± 2.64 ^a^	13.80 ± 1.62 ^a^
	W	23.81 ± 1.12 ^b^	6.49 ± 1.02 ^a^
10%	E	37.96 ± 14.96 ^b^	14.51 ± 0.82 ^a^
	W	25.64 ± 1.05 ^bc^	7.72 ± 0.52 ^a^
15%	E	43.47 ± 0.86 ^b^	34.16 ± 4.83 ^b^
	W	28.01 ± 1.68 ^c^	20.31 ± 2.72 ^b^
20%	E	51.08 ± 5.42 ^b^	60.93 ± 9.76 ^c^
	W	75.86 ± 1.42 ^d^	49.66 ± 2.60 ^c^
25%	E	111.31 ± 2.53 ^c^	111.59 ± 3.22 ^d^
	W	97.66 ± 0.95 ^e^	114.24 ± 9.12 ^d^
Part		ns	
Sample		***	
Solvent		ns	
Sample × part		***	
Part × solvent		ns	
Sample × solvent		***	
Sample × solvent × part		***	

Notes: 5–25% TNF addition; TE—Trolox equivalent; DM—dry matter, mean values with different lower case letters imply significant differences between the means in the columns at *p* < 0.05. Interaction graph for second-order interaction analysis ***—p < 0.001.

## Data Availability

The original contributions presented in this study are included in the article/Supplementary Materials. Further inquiries can be directed to the corresponding author.

## Supplementary Materials

The following supporting information can be downloaded at: https://www.mdpi.com/article/10.3390/foods14020229/s1, Figure S1. Photos of bread—0 days; Figure S2. Photos of bread—7 days.
