# Supplementary material for: Characteristics of Soft Wheat and Tiger Nut (Cyperus esculentus) Composite Flour Bread
Source: Foods. 2025 Jan 13;14(2):229. doi: 10.3390/foods14020229 (PMC11764678; doi:10.3390/foods14020229)
Supplement: Supplementary file 1 [file foods-14-00229-s001.zip › foods-3317328-supplementary.pdf]

foods

MDPI

100% wheat	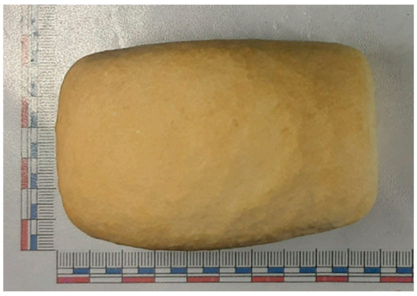	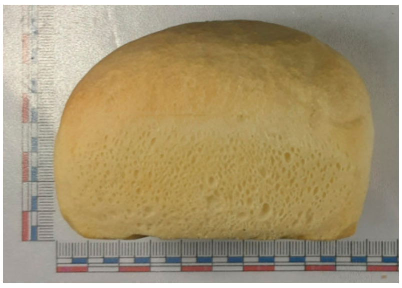	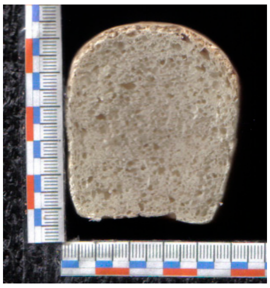
5%	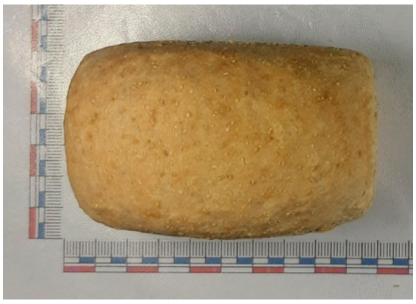	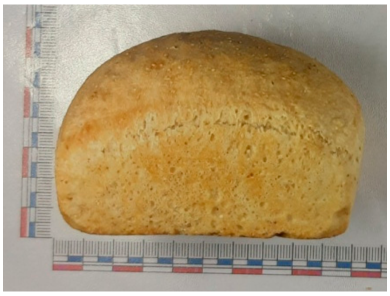	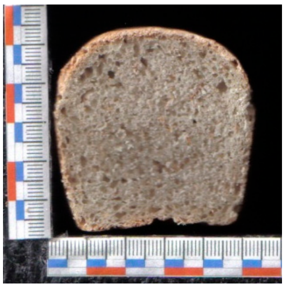
10%	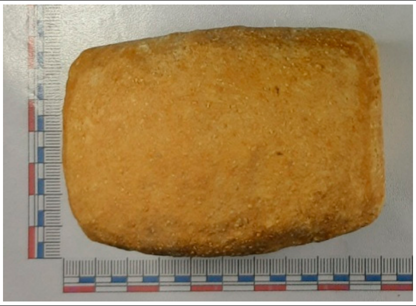	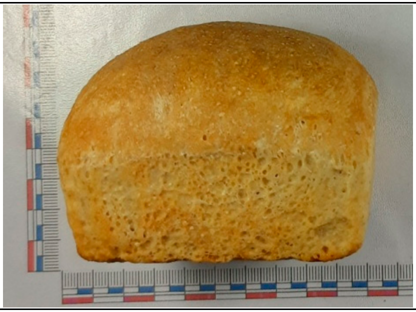	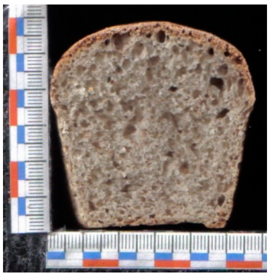
15%	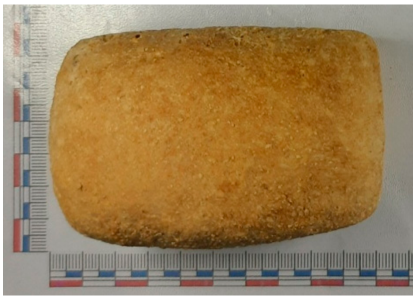	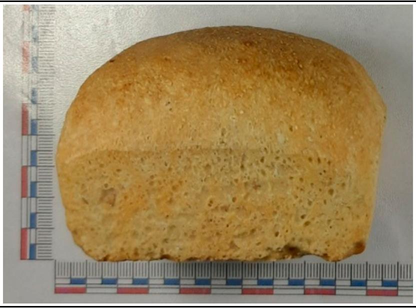	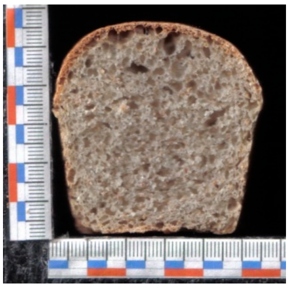
20%	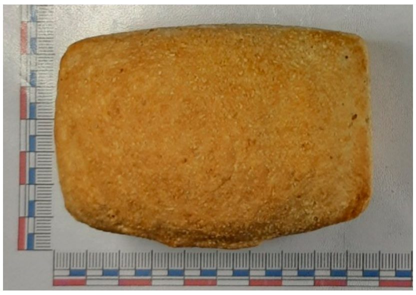	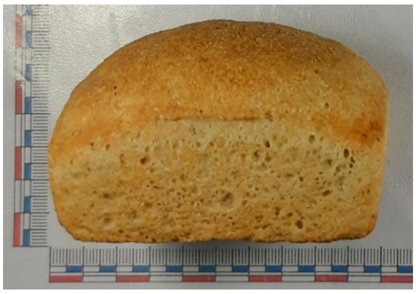	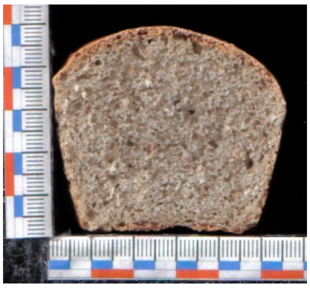

25%	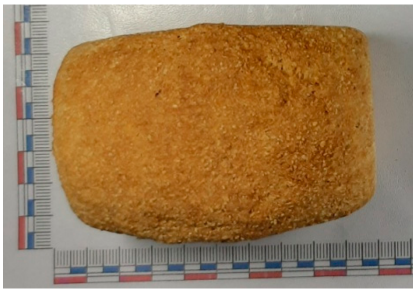	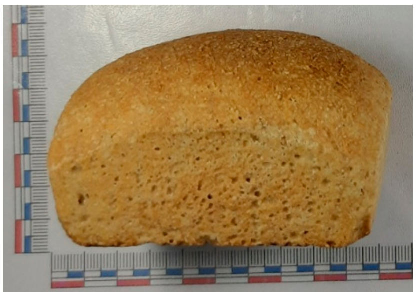	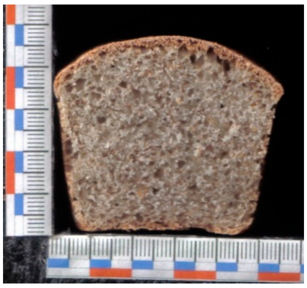

Supplementary Figure S1. Photos of bread—0 days.

100% wheat	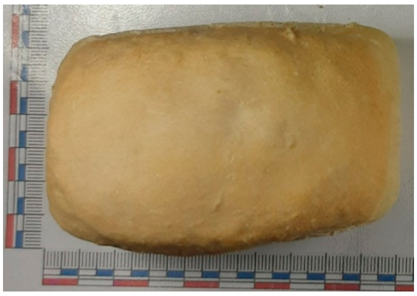	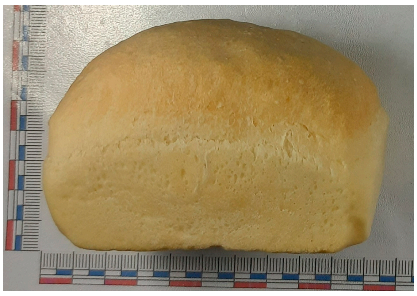	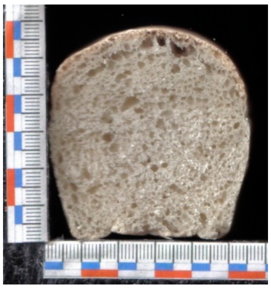
5%	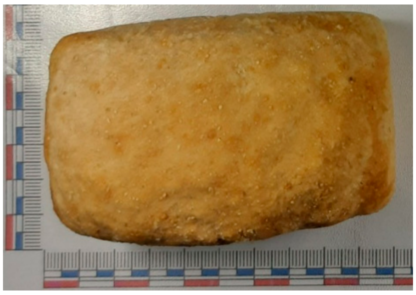	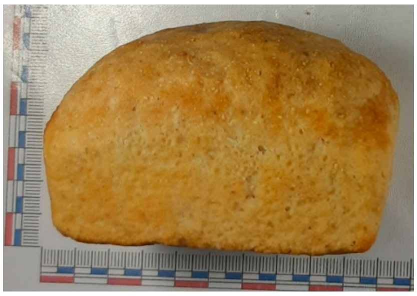	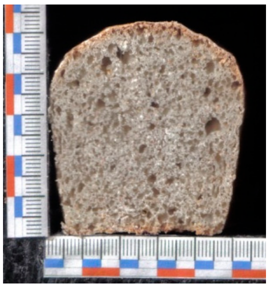
10%	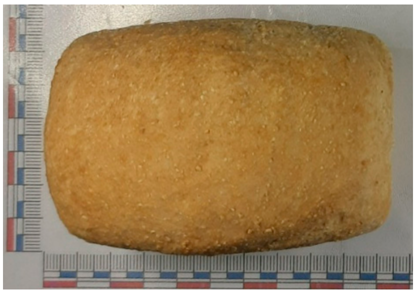	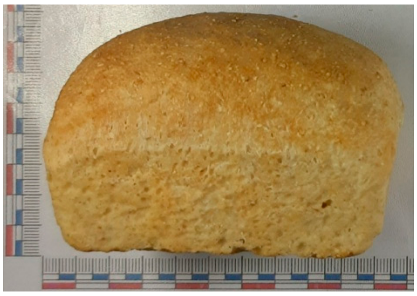	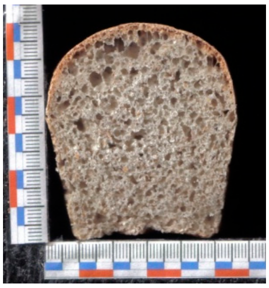
15%	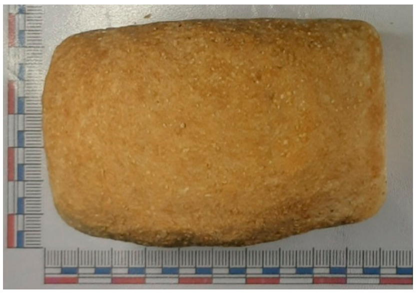	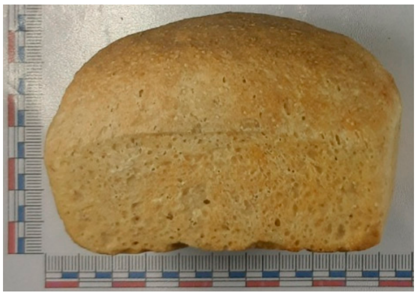	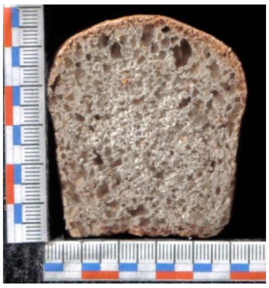

20%	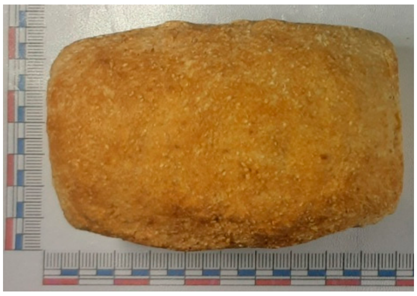	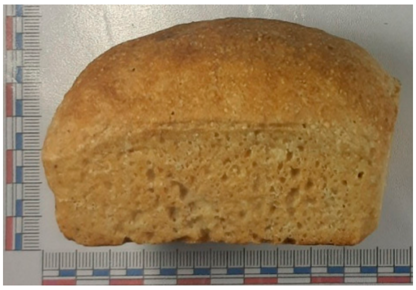	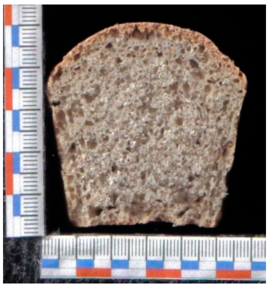
25%	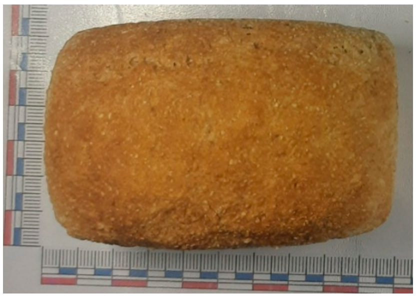	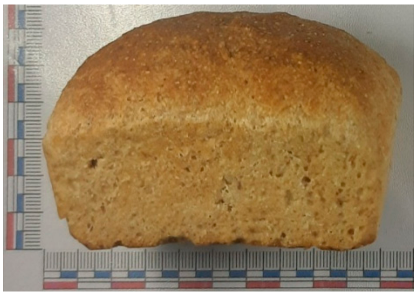	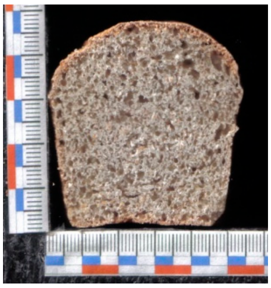

Supplementary Figure S2. Photos of bread—7 days.
